# DecoFungi: a web application for automatic characterisation of dye decolorisation in fungal strains

**DOI:** 10.1186/s12859-018-2082-9

**Published:** 2018-02-27

**Authors:** César Domínguez, Jónathan Heras, Eloy Mata, Vico Pascual

**Affiliations:** Department of Mathematics and Computer Science, University of La Rioja, Ed. CCT. C/ Madre de Dios 53, Logroño, 26006 Spain

**Keywords:** Fungal strains, Dye decolorisation, Image analysis, Deep learning, Transfer learning

## Abstract

**Background:**

Fungi have diverse biotechnological applications in, among others, agriculture, bioenergy generation, or remediation of polluted soil and water. In this context, culture media based on color change in response to degradation of dyes are particularly relevant; but measuring dye decolorisation of fungal strains mainly relies on a visual and semiquantitative classification of color intensity changes. Such a classification is a subjective, time-consuming and difficult to reproduce process.

**Results:**

DecoFungi is the first, at least up to the best of our knowledge, application to automatically characterise dye decolorisation level of fungal strains from images of inoculated plates. In order to deal with this task, DecoFungi employs a deep-learning model, accessible through a user-friendly web interface, with an accuracy of 96.5*%*.

**Conclusions:**

DecoFungi is an easy to use system for characterising dye decolorisation level of fungal strains from images of inoculated plates.

## Background

Fungi are important sources of metabolites and enzymes which have diverse biotechnological applications in agriculture; the food, paper, and textile industries; the synthesis of organic compounds and metabolites with pharmaceutical activities; cosmetic production; bioenergy generation; and remediation of polluted soil and water [1]. Because of the considerable diversity of fungal species, that are distributed in all ecosystems of the planet and occupy diverse niches as biotrophs or saprophytes, the isolation and characterisation of new strains with potential for biotechnological applications remains to be a dynamic field of mycological research.

In this context, isolation of fungal strains with biotechnological relevance, their identification, and their morphological and physiological characterisation is an important topic, for which selective media are routinely used for strain isolation and for detection of their extracellular metabolites or enzymes. To that aim, culture media based on color change, in response to degradation of dyes, are particularly relevant.

Most color-change assays rely on a visual and semiquantitative classification of color intensity changes, using an arbitrary scale for making comparative analyses between the different assayed fungal strains [2]. This approach implies that the results from assays are subjective, time-consuming, and unreproducible within the same laboratory and also across laboratories, even when assays are made under the same experimental conditions. Therefore, automatic and reliable tools for the selection and characterisation of fungal strains are needed for avoiding the dependence on the experimenter’s interpretation that is commonly present when assessing fungal capacity for dye decolorisation.

To tackle this problem, we have developed DecoFungi, a web application that employs computer vision and deep learning techniques for automatic characterisation of dye decolorisation in fungal strains.

## Implementation

The automatic characterisation of dye decolorisation level in fungal strains fits in the category of image-classification problems; a set of problems that can be undertaken by using different computer vision and machine learning techniques. Currently, the main methods employed for image-classification are deep-learning techniques [3]; and this is also the approach followed in DecoFungi.

DecoFungi employs a technique known as *transfer learning*, that consists in using the output of a deep neural network, trained in a source task, as “off-the-shelf” features to train a complete new classifier for the target task [4]. In particular, in DecoFungi, we use the *Resnet 50* neural network [5], trained in the ImageNet challenge, to extract features from images of fungal strains; and such features are employed to train a machine learning classifier. The choice of Resnet 50 was based on an exhaustive statistical study of different alternatives combining different source deep neural networks and machine-learning classifiers. Such a statistical analysis shows that the use of Resnet 50 can achieve an accuracy of 96.5*%*, see the following section.

DecoFungi provides 4 execution modes: analyse an image, analyse an image with its control image, analyse a zip file, and analyse a zip file containing a control image. In the first execution mode, the user must upload to DecoFungi an image of a Petri Dish containing a fungal strain. In the second mode, the user must provide, in addition to the image of the fungal strain, a control image of a sample containing only the employed dye — as it has been shown by our statistical study, this produces more accurate results. The latter two options — the zip-based execution modes — are based on the former and are a way to simplify the analysis of batches of images.

**Fig. 1:**
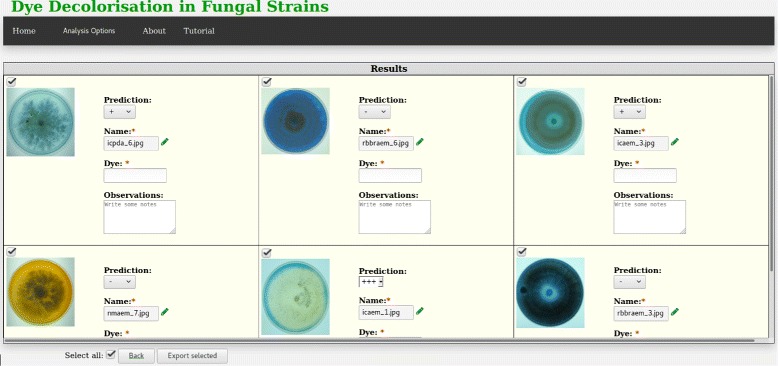
Graphical interface of DecoFungi showing the dye decolorisation level of several fungal strains

Independently of the execution mode, and to facilitate its usability and learnability, the results produced by DecoFungi are shown using always the same table — see Fig. 1. For each analysed fungal strain, DecoFungi provides the decolorisation level — using one of the following four labels: “-” (no decolorisation), “+”, “++”, and “+++” (completely decolorised) — the name of the image, the dye employed in the fungal strain, and some observations — the latter two fields are initially empty and can be filled by the user; and all of them can be modified. The results can be exported into an Excel file for further usage.

DecoFungi is implemented in Python using several open-source libraries: Django (as the Web application framework), OpenCV (library for image processing and computer vision), the Keras framework with a Tensorflow back-end (provides the deep learning techniques), and the scikit-learn library (library for machine learning).

## Results and discussion

A thorough comparative study was conducted to evaluate the performance of different models and decide which one was employed in our application. A total of 1204 images of dye decolorisation assays were analysed. The images of the dataset were annotated by biological experts with one of the following four labels indicating the decolorisation level: “-” (no decolorisation), “+”, “++”, and “+++” (completely decolorised). The dataset consists of 1204 images: 306 “-” images, 313 “+” images, 297 “++” images, and 288 “+++” images.

From the dataset of images, we use the transfer learning approach to extract features from images by considering the following 8 publicly available networks: DenseNet [6], GoogleNet [7], Inception v3 [8], OverFeat [9], Resnet 50 [5], VGG16 [10], VGG19 [10], and Xception v1 [11]. In all these networks, we consider two different approaches to generate the feature vector that describes an image. In the former, we extract the features from the image using the network, and that is its feature vector. In the latter, we stack the image with a control image of the dye; and, subsequently, the features are computed from the stacked image, and used as feature vector of the original image.

The feature vectors obtained using one of the previously mentioned approaches are fed to a classifier that is trained with them. The 6 classifiers that are considered in this work are Extremely Randomised Trees (from now on ERT) [12], KNN [13], Logistic Regression (from now on LR) [14], Multilayer Perceptron (from now on MLP) [15], Random Forest (from now on RF) [16], and Support Vector Machines (from now on SVM) [17]. The classification models produced by each combination of descriptor and classification algorithm are systematically evaluated by means of a statistical study using the methodology presented in [18, 19].

In order to validate the different classification models, a stratified 10-fold cross-validation approach was employed. To evaluate the performance of the classifiers, we measured their accuracy (i.e. the proportion of samples for which the model produces the correct output), the results are taken as the mean and standard deviation of the accuracy for the 10 test sets. The hyper parameters of each classification algorithm were chosen using a 10-fold nested validation with each of the training sets, and using a randomised search on the parameters distributions.

The results of this study are presented in Tables 1 and 2 showing that the best method, achieving an accuracy of 96.5*%*, is obtained when the control image is employed, Resnet 50 is used as network, and SVM is employed as classifier using the radial basis function (RBF) kernel. If a control image is not available, the best model is the one that combines Resnet 50 as network and LR as classifier — obtaining an accuracy of 94.5*%*. Since DecoFungi provides the functionality to analyse fungal strains both using and without using a control image, the two aforementioned models have been deployed in the web application (if the user does not provide a control image, the model that combines ResNet 50 and LR is applied; otherwise, the model that combines ResNet 50 and SVM is employed).

**Table 1:** Mean (and standard deviation) for the different studied models without considering the control image to generate the feature vectors

Network	ERT	KNN	LR	MLP	RF	SVM
DenseNet	*91.4(1.7)*	84.0(3.3)	90.1(2.5)	57.3(10.4)	87.3(2.1)	33.3(4.7)
GoogleNet	*92.4(2.1)*	49.2(3.8)	89.4 (2.4)	85.7 (5.9)	89.4 (2.1)	60.5(4.8)
Inception v3	88.6(2.8)	83.1(3.5)	*92.6(1.2)*	91.1(2.1)	80.0(2.7)	34.6(4.8)
OverFeat	89.5(2.5)	85.8(4.0)	91.2(2.5)	91.7(2.3)	85.8(4.0)	*92.5(2.3)*
Resnet 50	93.5(1.9)	46.4(4.9)	**94.5(1.7)**	93.3(2.7)	89.9(2.1)	73.1(6.1)
VGG16	89.9(2.3)	79.1(3.1)	*91.7(1.8)*	89.8(2.8)	82.5(2.2)	31.3(4.9)
VGG19	90.1(2.1)	84.4(3.1)	*92.7(2.3)*	90.9(2.4)	78.7(4.3)	33.1(4.7)
Xception v1	90.1(2.7)	87.8(2.9)	*93.5(1.6)*	92.2(2.0)	82.1(3.7)	91.9(1.3)

The best result for each network in *italics*, the best result in **bold** face

**Table 2:** Mean (and standard deviation) for the different studied models considering the control image to generate the feature vectors

Network	ERT	KNN	LR	MLP	RF	SVM
DenseNet	*96.2(2.3)*	85.5(4.3)	94.3(2.8)	62.2(18.6)	93.9(2.9)	42.5(4.6)
GoogleNet	92.5(3.1)	88.6(2.5)	92.4(2.8)	92.0(3.1)	88.6(4.2)	*95.4(2.2)*
Inception v3	93.0(2.7)	87.6(3.1)	*95.5(1.6)*	94.3(2.1)	86.8(2.0)	46.4(4.8)
OverFeat	87.2(2.4)	82.6(4.5)	92.7(2.1)	92.2(2.6)	82.0(3.5)	*93.0(2.4)*
Resnet 50	92.6(2.8)	90.1(3.2)	95.2(2.3)	94.7(2.3)	89.6(1.8)	**96.5(1.6)**
VGG16	*95.0(2.0)*	86.4(2.3)	94.7(1.7)	92.4(1.7)	89.2(3.5)	33.1(4.3)
VGG19	94.4(1.6)	84.5(2.9)	*94.6(2.3)*	92.4(2.7)	87.1(2.3)	33.7(4.4)
Xception v1	93.5(2.7)	89.9(4.4)	*95.2(2.3)*	94.8(1.7)	86.8(3.0)	94.8(1.9)

The best result for each newtork in *italics*, the best result in **bold** face

## Conclusion

DecoFungi is the first web application to easily and automatically predict the dye decolorisation level in fungal strains. The use of DecoFungi greatly reduces the burden and subjectivity of visually classifying the dye decolorisation level by providing a standard and reproducible method with high accuracy.

In the future, and to better relieve the problem of subjective judgement, we will evaluate the decolorisation level based on modelling of fungal strain images rather than expert labelling. In addition, we plan to study whether it is possible to move from the discrete measure (that takes the value of “-”, “+”, “++”, or “+++”) of decolorisation level to a more informative continuous measure that still remains to be defined.

## Availability and requirements

- Project name: DecoFungi.
- Project home page: http://www.unirioja.es/decofungi.
- Source code:
- Operating system(s): Platform independent.
- Programming language: Python.
- Other requirements: None.
- License: GNU GPL v3.
- Any restrictions to use by non-academics: restrictions specified by GNU GPL v3.

DecoFungi does not require installation, it can be run in any browser.

## References

[1] Chambergo FS, Valencia EY (2016). Fungal biodiversity to biotechnology. Appl Microbiol Biotechnol.

[2] Sorensen A (2011). Onsite enzyme production during bioethanol production from biomass: screening for suitable fungal strains. Appl Biochem Biotechnol.

[3] Krizhevsky A, Pereira F, Burges CJC, Bottou L, Weinberger KQ (2012). Advances in Neural Information Processing Systems 25. ImageNet Classification with Deep Convolutional Neural Networks.

[4] Pan SJ, Yang Q (2010). A survey on transfer learning. IEEE Trans Knowl Data Eng.

[5] He K (2016). Deep Residual Learning for Image Recognition. Proceedings of IEEE Conference on Computer Vision and Pattern Recognition (CVPR’16).

[6] Huang G, Liu Z, van der Maaten L, Weinberger KQ (2017). Densely connected convolutional networks. Proceedings of the IEEE Conference on Computer Vision and Pattern Recognition (CVPR’17)..

[7] Szegedy C (2015). Going deeper with convolutions. Proceedings of IEEE Conference on Computer Vision and Pattern Recognition (CVPR’15), IEEE Computer Society.

[8] Szegedy C, et al. Rethinking the Inception Architecture for Computer Vision. CoRR. 2015;:abs/1512.00567. http://arxiv.org/abs/1512.00567.

[9] Sermanet P, et al. OverFeat: Integrated Recognition, Localization and Detection using Convolutional Networks. CoRR. 2013;:abs/1312.6229. http://arxiv.org/abs/1312.6229.

[10] Simonyan K, Zisserman A. Very Deep Convolutional Networks for Large-Scale Image Recognition. CoRR. 2014;:abs/1409.1556. http://arxiv.org/abs/1409.1556.

[11] Chollet F. Xception: Deep Learning with Depthwise Separable Convolutions. CoRR. 2016;:abs/1610.02357. http://arxiv.org/abs/1610.02357.

[12] Geurts P, Ernst D, Wehenkel L (2006). Extremely randomized trees. Mach Learn.

[13] Cover T, Hart P (2006). Nearest Neighbor Pattern Classification. IEEE Trans Inf Theor.

[14] McCullagh P, Nelder JA (1989). Generalized Linear Models.

[15] Bishop CM (1995). Neural Networks for Pattern Recognition.

[16] Breiman L (2001). Random Forests. Mach Learn.

[17] Cortes C, Vapnik V (1995). Support-Vector Networks. Mach Learn.

[18] Garcia S (2010). Advanced nonparametric tests for multiple comparisons in the design of experiments in computational intelligence and data mining: Experimental analysis of power. Inf Sci.

[19] Sheskin D (2011). Handbook of Parametric and Nonparametric Statistical Procedures.

